# Bottom-up and top-down paradigms of artificial intelligence research approaches to healthcare data science using growing real-world big data

**DOI:** 10.1093/jamia/ocad085

**Published:** 2023-05-15

**Authors:** Michelle Wang, Madhumita Sushil, Brenda Y Miao, Atul J Butte

**Affiliations:** Bakar Computational Health Sciences Institute, University of California, San Francisco, San Francisco, California, USA; Bakar Computational Health Sciences Institute, University of California, San Francisco, San Francisco, California, USA; Bakar Computational Health Sciences Institute, University of California, San Francisco, San Francisco, California, USA; Bakar Computational Health Sciences Institute, University of California, San Francisco, San Francisco, California, USA; Department of Pediatrics, University of California, San Francisco, San Francisco, California, USA

**Keywords:** artificial intelligence computational methods, real-world data, electronic health records

## Abstract

**Objectives:**

As the real-world electronic health record (EHR) data continue to grow exponentially, novel methodologies involving artificial intelligence (AI) are becoming increasingly applied to enable efficient data-driven learning and, ultimately, to advance healthcare. Our objective is to provide readers with an understanding of evolving computational methods and help in deciding on methods to pursue.

**Target Audience:**

The sheer diversity of existing methods presents a challenge for health scientists who are beginning to apply computational methods to their research. Therefore, this tutorial is aimed at scientists working with EHR data who are early entrants into the field of applying AI methodologies.

**Scope:**

This manuscript describes the diverse and growing AI research approaches in healthcare data science and categorizes them into 2 distinct paradigms, the bottom-up and top-down paradigms to provide health scientists venturing into artificial intelligent research with an understanding of the evolving computational methods and help in deciding on methods to pursue through the lens of real-world healthcare data.

## INTRODRUCTION

The synergies between the fast-growing real-world health data and the recent rapid advancement in computational sciences and artificial intelligence (AI) are changing how we understand and can improve healthcare. Real-world data are defined as “the data relating to patient health status and/or the delivery of health care routinely collected from a variety of sources,”^1^ such as claims data from payers, electronic health records (EHRs) data from healthcare systems, patient-reported data from registries, and the real-time continuous streams of data generated by wearable technologies.^2^ Since these data detail individual patients’ health statuses, medical interventions received, trajectory or progression of diseases, and patients’ experiences, they represent unique sources of information with which to improve healthcare, largely through complementing clinical trials, supporting regulatory decision-making, and optimizing or personalizing patient care.^3–5^

**Figure 1.**
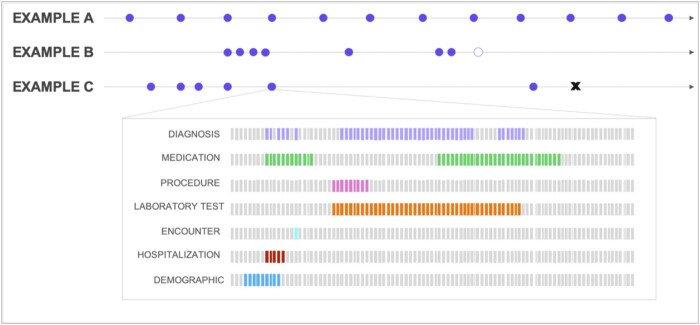
Sample data to show EHR data characteristics. Examples A, B, and C are hypothetical patient encounters with the healthcare system. The filled circles indicate any encounter with the healthcare system including visits, hospitalizations, or telemedicine visits. The empty circle indicates a patient that stopped receiving services from the healthcare system. The “X” marks the deceased status of the patient. Example A shows that the patient had regular encounters with the provider or healthcare system over time, while examples B and C are patients that have irregular encounters over time and across different lengths of time. For each encounter (filled circle), there may be different types of EHR data recorded. For instance, as shown in the inset figure above, there could commonly be diagnosis, medication, and procedure codes and hospitalization information (if applicable) being recorded for each encounter. In addition, laboratory tests and results are often available as well as patient demographics. In the inset figure, the bars symbolize the high dimensionality of EHR data—each bar indicates 1 code or data for each type of EHR data. The highlighted bars indicate the specific codes or data recorded during 1 encounter for 1 particular patient. EHR: electronic health record.

EHR data are a type of real-world data that are particularly enriched with comprehensive patient-level clinical details. Since the adoption of EHR systems worldwide, EHR data have become a fast-growing, abundant, and ubiquitous real-world source to study continuous longitudinal patient health and healthcare systems themselves. EHR data comprise different modalities, including unstructured data (eg, imaging, text, and video) and structured data, and have distinctive characteristics, such as irregular time series, high dimensionality, longitudinal nature, incompleteness or missingness, and low-resource characteristics (Figure 1). Irregular time series refers to how patients may receive clinical care at varying time intervals, which would then lead to clinical data (eg, different laboratory test results and other measurements) being captured at different frequencies; high-dimension and longitudinal nature describe the possibility of patients with complex diseases and long histories of care received within the same healthcare system; incompleteness or missingness in data arises from patients who are no longer with the same healthcare system, data not reported nor observed due to the lack of a medical reason for those measurements, or just simply missing at random; low-resource refers to the significant lack of gold standard annotated data sets available as these data are particularly labor intensive and costly to generate in highly specialized medical domains.

Due to their unique characterization and increasing complexity as they accumulate, EHR data can be challenging to study using traditional methods involving manual reviews or statistical methods. The advancement of AI computational methods with high computing power and novel algorithms to process complex big data have led to a rapidly developing research field intersecting AI and healthcare.^6–8^ However, healthcare scientists who are early entrants into the field of applying computational methods to their research may find it challenging due to its rapidly developing and diverse methods. Moreover, growing healthcare big data warrant attention and discussion on ways to better utilize it efficiently and effectively. Therefore, this review aims to provide health scientists interested in applying AI computational methods to the study of real-world data with an understanding of relevant current research approaches, which we have categorized into top-down and bottom-up paradigms. Furthermore, we propose a decision-helping tool to help health scientists identify suitable computational methods to pursue. We begin by introducing building-block concepts such as types of machine learning, classical machine learning algorithms, deep learning neural networks, and techniques useful in low-resource data followed by the respective discussions of the top-down and bottom-up paradigms in AI computational methods, their applications and limitations, and emerging research opportunities. Lastly, we provide a table of example programs focusing on AI computational methods in healthcare as a resource for health scientists learn in-depth knowledge and technical skills.

## BUILDING-BLOCK CONCEPTS

### Machine learning: supervised, unsupervised, and semisupervised learning

Supervised learning refers to training (fitting) algorithms, or models, using data annotated with ground truth labels (gold standard data).^9^ These ground truth labels in EHR data require clinically trained individuals equipped with domain knowledge to be generated. Hence, EHR data remain largely unlabeled and unannotated, and it can be labor intensive, costly, and time consuming to create ground truth labels for sufficient amounts of training data. In contrast to supervised learning, unsupervised learning refers to situations when algorithms are used to recognize patterns in data without any ground truth labels.^9^ This approach is often used to identify subgroups in data. Semisupervised learning is a hybrid approach in which algorithms are trained using only small amounts of gold standard-labeled data while the rest of the data remain unlabeled or with “silver-standard” labels that may be easily obtained but at a lower quality compared to gold standard labels.^10^ EHR data particularly benefit from semisupervised learning due to its low-resource nature.

### Classical machine learning algorithms

Classical machine learning algorithms are a set of data-driven algorithms that can fit data based on statistical and mathematical rules.^9^^,^^11^ Training classical machine learning algorithms often requires feature engineering, which is a preprocessing step that can involve data transformation and selection of important features (covariates) based on domain knowledge. Feature engineering may improve the performances and interpretability of the resulting machine learning models. Compared with advanced algorithms such as artificial neural networks, machine learning algorithms are generally more interpretable and require much less computational resources and data to train. Machine learning algorithms commonly applied in the medical domain include regressions, support vector machines, random forests, and many others.

### Artificial neural networks

In contrast to the classical machine learning algorithms, artificial neural networks are deep learning algorithms consisting of multiple (deep) layers of neural networks which, through learning layers of nonlinear functions, allow for better representation learning and pattern recognition of input data.^12^ Minimal human-involved feature engineering is required, making deep learning algorithms more suitable for high-dimensional and longitudinal data than classical machine learning algorithms. Furthermore, with multiple layers of neural networks, deep learning algorithms, compared with classical machine learning algorithms, may be better able to study data characterized with complex representations, such as EHR data. However, deep learning algorithms generally require much larger magnitude of datasets and computational resources. The deep learning algorithms commonly applied in the medical domains include convolutional neural networks for image processing, transformer-based models for clinical texts, and recurrent neural networks for sequential data.^13–16^

### Evolving techniques for low-resource data: transfer learning and semisupervised learning

In low-resource data like EHR data where gold standard labels do not exist or are difficult to obtain, techniques that leverage available unlabeled data can be very helpful. Transfer learning has been gaining popularity in the healthcare domain in recent years.^17^^,^^18^ This methodology involves learning generalizable patterns from source domain data that remained unlabeled during the initial training phase, termed pretraining.^19^ Subsequently, the algorithm, leveraging the previously learned generalizable knowledge, is thereby fine-tuned to perform separate but related tasks in the target domain data. Its applications to EHR big data have been important for studying unstructured data, including images and clinical texts.

Semisupervised learning, particularly approaches to integrate silver-standard labels and self-supervised learning, has also been increasingly applied in the health data science in recent years. For instance, EHR structured data and the concepts of unified medical language systems have been used as silver-standard labels in research involving clinical notes that are unstructured and unlabeled.^20^^,^^21^ Self-supervised learning is another increasingly used technique, commonly used during the “pretraining” phase.^22^ It can be considered a type of semisupervised learning as the algorithms are trained with data without human-involved annotations. Instead, the labels are automatically generated by manipulating the data themselves. For instance, predicting the rotation angle of rotated images or the masked words in a sentence. This allows the algorithms to learn high-level representations of the data and is often applied during the pretraining phase in transfer learning, which can jumpstart other downstream training.^23^

**Figure 2.**
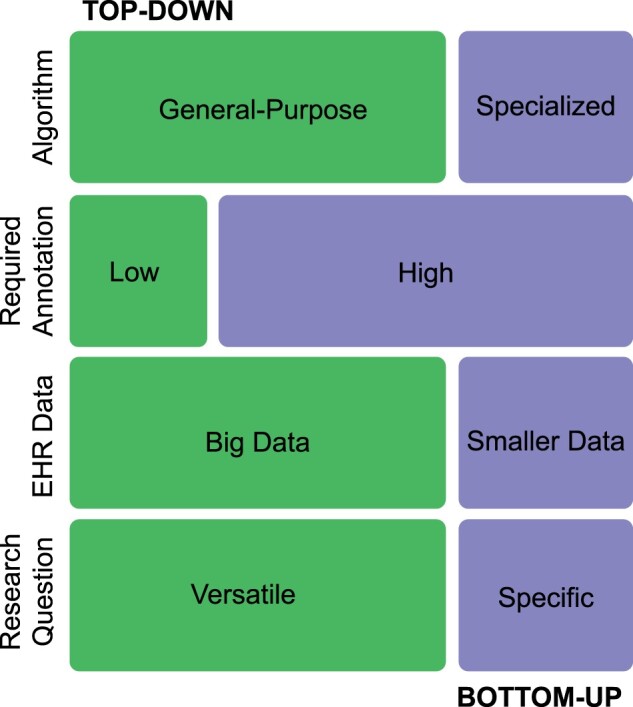
Comparing bottom-up and top-down learning approaches. The bottom-up approaches are driven by specific research questions, require annotated data, and the resulting algorithm is specialized for specific tasks (eg, predicting prolonged hospitalization, phenotype classification). In contrast to bottom-up approaches, the top-down paradigm starts from developing a general-purpose algorithm that requires minimal or no annotation and utilizes big EHR data. The resulting algorithm is versatile (nonspecific) and can be further fine-tuned to perform specialized tasks. EHR: electronic health record.

The building-block concepts above introduced machine learning frameworks, classical machine learning, neural network algorithms, and techniques particularly relevant to low-resource healthcare data. Generally, AI computational methodologies comprise combinations of these building blocks at different stages where applicable. Specifically, the availability of computational resources, the complexity of the research questions and the data, and whether a sufficient amount of training data with or without accessible gold standard labels is available are important considerations when developing computational methods. We categorized the common major AI computational methods in the healthcare domain into top-down and bottom-up paradigms based on the data and annotation needed, training technique, and function of the resulting algorithm (Figure 2).

## BOTTOM-UP PARADIGM

There have been a number of prior studies summarizing the evolving landscape surrounding real-world EHR data, but the majority focused on the bottom-up paradigm of AI computational methods.^24–29^ The bottom-up paradigm approach refers to a situation where a computational tool, method, or algorithm is trained from scratch specifically to address a particular research question involving only “small data,” meaning a portion of the entire EHR big data (Figure 2). However, all training data included require ground truth labels, and the resulting algorithm is specialized in 1 specific task (Figure 2).

**Figure 3.**
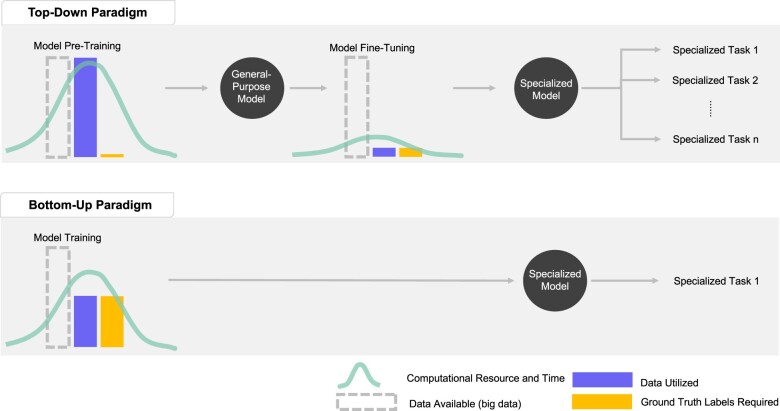
Algorithm-training process for top-down and bottom-up paradigms. The top-down paradigm utilizes EHR big data available without the need of ground truth labels during the pretraining phase, which can be computationally demanding. The resulting algorithm is a general-purpose model that can be fine-tuned to specialize in specific tasks. The fine-tuning phase requires only a small fraction of the available data to have ground truth labels for supervised training. The computational resource needed for model training during the fine-tuning phase is significantly less than the pretraining phase. The bottom-up paradigm approaches do not involve pretraining and fine-tuning phases as described in the top-down paradigm approaches. In contrast, the bottom-up paradigm approaches utilize “smaller data,” which are the data relevant to the research question of interest. In addition, all the data involved in model training typically require ground truth labels for supervised training. The resulting model is then specialized for a specific task or research question. The computational resource needed may be less than the pretraining phase in the top-down paradigm but may increase as model complexity increases. EHR: electronic health record.

**Figure 4.**
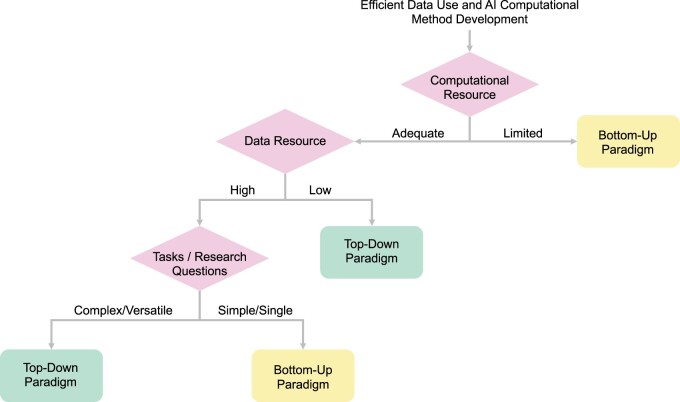
A Decision-making flow-chart for efficient data use and AI method development. To decide on suitable computational methods to pursue, healthcare scientists may consider 3 key factors: availability of the computational resource, data resource, and the complexity of the research questions itself. When computational resource is limited, the bottom-up approach may be more suitable, as top-down paradigm approaches are computationally demanding. When computational resource is adequate but data resource is low, top-down paradigm approaches are worth considering. When data resource is low, that is, low-resource data and only a very limited amount of data with ground truth labels is available, methods such as transfer learning in the top-down approach that leverage other unlabeled data are ideal. However, when both computational resource and data availability are high, the research question is complex, or versatile algorithms are required to perform multiple tasks, the top-down paradigm may be superior. If the research question is relatively straightforward and the algorithm is performing single or very few tasks, the bottom-up paradigm approach may be more efficient.

In contrast to the top-down paradigm, bottom-up paradigm approaches only utilize a subset of the available EHR data and do not involve pretraining or fine-tuning processes during model training (Figure 3). As a result, this approach is particularly useful when computational resources are limited as the pretraining phase in top-down approaches can be significantly more computationally demanding. Furthermore, bottom-up approaches can be more efficient when the research questions involve simpler or few tasks and adequate computational resources as well as sufficient training data with ground truth labels are available (Figure 4).

Applications of bottom-up paradigm approaches in medicine have been predominately centered around developing predictive models to forecast new onsets or recurrences of disease, disease progressions or trajectories, adverse event developments, and outcomes such as hospitalizations or mortality.^30–41^ These predictive models identify patients likely to develop undesirable outcomes and allow clinicians to provide timely care or interventions, in turn optimizing healthcare for individual patients.

Another major area of AI research is focused on developing algorithms to support the classification or identification of phenotypic disease characteristics embedded in EHR.^40–45^ Such applications can be useful for supporting safety surveillance of therapeutics or monitoring for potential secondary complications in patients. Other research efforts involve comparing various computational methods including classical machine learning, deep learning artificial neural networks, and statistical approaches (eg, conventional survival models) or building universal frameworks to help streamline the application of computational algorithms to real-world clinical data.^46–49^ In the realm of unstructured EHR data, prominent examples include convolutional neural networks that work on medical imaging (eg, recognizing diabetic retinopathy), recurrent neural networks that work on sequential time series data such as electrocardiograms, speech decoding in paralyzed patients, and machine learning-based or recurrent neural networks to study clinical notes.^13–16^^,^^50^

While the bottom-up approaches may seem more straightforward and faster to develop, they can be limited when the research questions are complex or involve multiple tasks to complete, which are not uncommon in healthcare research using real-world EHR data. For instance, there are 15 adverse events of special interest that may be associated with novel COVID-19 vaccines.^51^ An algorithm that can identify all 15 adverse events would be much more efficient than training single models to identify each type of adverse event. However, the complexity of algorithms increases as the difficulty of the research questions increase, and such advanced algorithms would require significantly more training data as well as ground truth labels for sufficient generalized training. Furthermore, sufficient amounts of training data often do not exist or may be extremely difficult to obtain from EHR data. As a result, there is a trade-off between the complexity of the algorithm needed to address a research question and the amount of obtainable training data and ground truth labels. The trade-off could be even more apparent in models involving multimodal EHR data (structured data, texts, and images). Lastly, making advanced models explainable and interpretable may also become more challenging as the complexity of the algorithm increases.

Another key shortcoming of bottom-up approaches relates to utilization of the available data resource. While vast amounts of EHR data are available and growing continuously, the bottom-up approach may be inefficient as it only utilizes a subset of data—for instance, a cohort of patients with a particular disease of interest—for algorithm training while leaving the rest of the EHR data unused. This can be considered an inefficient use of data resource as the rest of the EHR big data, although not directly related to a specific patient cohort of interest, still contains comprehensive clinical data representing clinical practices and medical knowledge that are generalizable and transferable. Therefore, approaches that allow learning from details embedded in the rest of the EHR big data could support more efficient downstream algorithm training and better utilization of available data resources.

## TOP-DOWN (REPRESENTATION LEARNING) PARADIGM

The top-down representation learning approach describes a strategy involving vast amounts of data from the source domain to create a general-purpose tool, method, or algorithm that is “versatile,” meaning it is not yet specialized like algorithms developed under the bottom-up paradigm but can be further fine-tuned to address specific unmet research (medical) needs (Figures 2 and 3). In contrast to bottom-up approaches where only a subset of EHR data would be needed for algorithm training, top-down approaches require significantly more data, all the EHR big data available, during the pretraining phase, but only a much smaller amount of data with ground truth labels would be needed during the fine-tuning phase (Figure 3).

Transfer learning, as explained above, is an exemplary top-down learning approach. When using the self-supervised learning technique during the pretraining phase using EHR big data as the source, the algorithm is trained to learn generalizable fundamental knowledge agnostically across diseases and therapeutic areas in medicine. Such fundamental knowledge can help “jumpstart” other relevant downstream trainings. The resulting algorithm is general-purpose and can be *efficiently* trained to specialize in specific research questions through fine-tuning using several-orders-of-magnitude less data compared to bottom-up paradigm approaches (Figure 3). However, computational resource is often more demanding for top-down approaches compared with bottom-up approaches owing to the pretraining phase, where a large amount of big data is required. However, top-down approaches are worth considering when diverse tasks or research questions are of interests, adequate computational resource is available, but only very limited training data with ground truth labels are available (Figure 4).

Such top-down representation learning approaches combining transfer learning and self-supervised learning techniques have been widely adopted in medical imaging applications using convolutional neural networks (Table 1).^22^^,^^52^^,^^53^ In recent years, they have also been applied in natural language processing, where large language models were trained on vast numbers of documents and then fine-tuned to perform a variety of downstream tasks including document classification, name entity recognition, relation extraction, summarization, and natural language understanding. The most widely adopted breakthrough application in medicine was the transformer-based models including bidirectional encoder representations from transformer (BERT) and the recent popular generative pretrained transformers (GPT).^54^^,^^55^ Several BERT models have been pretrained using the unstructured EHR text data clinical notes, and they have achieved marked improvement in a variety of downstream tasks including supporting patient outcome prediction and phenotype detection using only very limited training data (Table 1).^56–59^

**Table 1.** Examples of top-down approaches with transfer learning and self-supervised pretraining strategies using EHR data

Author, year	Model name	Model adopted	EHR data type	Approximate data volume	Few-shot learning capability
Azizi et al, 2021^52^	MICLe	ResNet	Images	224,000 images	NA
Sowriraran et al, 2021^53^	MoCo-CXR	ResNet	Images	224,000 images	Yes
Alsentzer et al, 2019^56^	Publicly Available Clinical BERT	BERT	Text	2 million documents	NA
Li et al, 2019^59^	EhrBERT	BERT	Text	1.5 million documents	NA
Huang et al, 2020^57^	ClinicalBERT	BERT	Text	2 million documents	NA
Zhang et al, 2020^58^	BERT-XML	BERT	Text	7.5 million documents	NA
Shang et al, 2019^63^	G-BERT	BERT	Structured data	30,000 distinct patients	NA
Li et al, 2020^61^	BEHRT	BERT	Structured data	1.6 million distinct patients	NA
Rasmy et al, 2021^60^	Med-BERT	BERT	Structured data	20 million distinct patients	Yes
Pang et al, 2021^62^	CEHR-BERT	BERT	Structured data	2.4 million distinct patients	Yes
Park et al, 2022^64^	MedGTX	BERT	Text and structured data	40,000 million distinct patients	NA

*Abbreviation*: EHR: electronic health record.

Top-down paradigm approaches have also been extended to structured EHR data to learn contextualized representations of medical codes (eg, diagnosis, medication, and laboratory test codes) assigned chronologically to individual patients during each clinical encounter using BERT-based algorithms (Table 1).^60–64^ The pretrained algorithm using structured EHR data with a standardized data model is a general-purpose model that can be easily shared with researchers from other institutions and is much more accessible and generalizable compared with bottom-up paradigm algorithms or algorithms involving unstructured EHR data. Furthermore, it may have the potential to achieve few-shot learning, where very few training data are needed during the fine-tuning training phase (Table 1). This is crucial in addressing the low-resource challenges in the medical domain.

Transfer learning has shown marked improvements in efficiency and model performance across various domains and data modalities, including both unstructured and structured EHR data. However, this strategy may be limited in terms of 3 key aspects: first, the source and target domains are best related to each other; otherwise, negative transfer can arise, leading to suboptimal performances of tasks in the target domain.^19^ Second, due to the large scales of data required during the pretraining phase, computational costs and time can be significantly more demanding compared with those for algorithms developed following the bottom-up paradigm. Third, the implementations and algorithm development could be much more complex and challenging, which would require highly trained individuals to perform.

## FUTURE OUTLOOK

Real-world EHR data are predicted to play an integral part in advancing healthcare across drug development, regulatory decision-making, and clinical care. As the amount of EHR data continue to grow exponentially, it is increasingly in demand for advanced AI computational methods to help unravel the intricate relationships between individual patient health statuses, diseases, as well as medical interventions and responses. In the last decade, the influx of applying AI computational methods to EHR data has led to successes in impacting clinical care and improving patient outcomes. However, the majority of them employed bottom-up approaches, which could be limited by generalizability, insufficient training data, and inefficient or underuse of available data. Transfer learning coupled with self-supervised learning techniques, a top-down approach, provides a framework to address these key shortcomings by creating a more general-purpose algorithm providing knowledge in medicine and clinical practices through learning from all the available EHR data. Such general-purpose algorithms can then be fine-tuned to specialize in diverse sets of downstream tasks with the potential capability of few-shot learning, meaning that they require significantly smaller training datasets (Figure 3).

While top-down learning may seem like a rational approach given the growing EHR big data, there are also scenarios where bottom-up approaches may be more suitable. To help health scientists interested in applying AI computational methods, we have developed an easy decision tool to help them decide if the bottom-up approach is sufficient or whether their research questions would benefit from the top-down paradigm approach (Figure 4). The decision tool is focused on computational resource, the availability of sufficient training data and ground truth labels, as well as the complexity of the computational tasks or research questions. Other factors, such as the level of training and experience the healthcare scientist has in computational research, are worth considering as the bottom-up paradigm may be simpler to implement than top-down approaches. Additionally, the availability of the pretrained models and if the resulting model is for research or real-world applications may be important deciding factors as well. The computational resources needed could be drastically reduced when working with an existing pretrained model and the implementation of its downstream applications could also be easier. However, deploying larger models to real-world applications (eg, integration into clinical workflow) may be impractical due to the associated high computational costs. Furthermore, if the interpretability of the algorithm is of priority, researchers may consider starting with bottom-up approaches with parsimonious algorithms (eg, classical machine learning algorithms), which could be easier to understand and consequently gain acceptance in the medical community. However, it is also important to recognize that models with high performances may have better utility and potential in improving healthcare even if it is not fully interpretable.^65^

This discussion introduced real-world EHR data, addressed the commonly used bottom-up paradigm and highlighted how top-down learning can be effective and efficient in fully utilizing the big EHR data available. Although we put slightly more emphasis on the use of structured EHR data owing to its improved accessibility, much patient-level data pertinent to patient health is only available as unstructured EHR data, such as images or clinical text. For instance, the results of a brain MRI and prior treatment exposures may only be found in image formats or documented in clinical notes. Hence, methods that can efficiently integrate multimodal data in EHR may be increasingly vital to advance the field toward learning holistic and deep representations of patients efficiently.^64^^,^^66–68^ Furthermore, while AI research is rapidly developing, we only discussed the commonly used AI computational methods and approaches that have been translated to the medical realm. Other advanced algorithms worth noting include reinforcement learning and generative adversarial networks, which have been applied in creating decision support tools and drug discovery research.^69^^,^^70^ Reinforcement learning in particular may find increasing applications in medicine to develop tools to identify or suggest personalized medical interventions tailored to individual patients’ unique health statuses and trajectories.^69^^,^^71–73^ Most excitingly, large language models such as the recently released ChatGPT, InstructGPT, GPT-4, and LLaMA are powerful models capable to perform complex natural language processing tasks and their potential applications in the field medicine are gaining high popularity rapidly^74–77^. Lastly, we also focused largely on supervised learning, whereas unsupervised learning has also been widely used in discovering potential novel phenotypes using EHR data.^40^^,^^41^^,^^78^^,^^79^

In summary, as real-world EHR data, which encode comprehensive patient-level data for health statuses, disease progression, treatment intervention, and patient experiences, continue to grow exponentially, methodologies and strategies to efficiently learn from and utilize the wealth of knowledge embedded in the data are becoming crucial in advancing healthcare. In the present review, we introduced basic concepts in computational methods and highlighted the advantages and limitations of developing AI methods following bottom-up or top-down representation learning paradigms. Our decision-making tool enables health scientists interested in engaging in computational research and real-world data to choose more efficient and suitable approaches to pursue. Furthermore, example programs and courses focusing on gaining in-depth understanding across topics including fundamental concepts in clinical data, AI computational methods and applications in research as well as real-world implementation have been provided to help health scientists venture into the field of AI and medicine (Table 2).

**Table 2.** Example courses and training programs focusing on AI computational methods in healthcare across research-based development to real-world applications

Program name	Sponsoring organizations	Course and program summary
Introduction to Biomedical and Health Informatics^80^	American Medical Informatics Association, Oregon Health and Science University	Gain broad understanding across implementation and development of informatics solutions for healthcare challenges. Course covers topics including electronic health records, data standard and interoperability, and novel technologies such as machine learning and blockchain.
Clinical Decision Support^81^	American Medical Informatics Association, University of Utah	Gain in-depth knowledge and state-of-the-art principles as well as practices to develop effective clinical decision support tools, standard, implementation, and evaluation.
Artificial Intelligence in Healthcare^82^	Stanford University	Gain fundamental understanding of clinical data, machine learning, and evaluation of AI applications in healthcare.
Medical AI Bootcamps^83^	Harvard University, Stanford University	Participate in team-based research projects, gain cutting-edge knowledge in AI medical research and machine learning technical skills.
Artificial Intelligence in Healthcare^84^	Massachusetts Institute of Technology	Gain comprehensive understanding of the growing roles of AI computational methods in health care focusing on real-world applications of AI in health care management and optimization.
Innovation with AI in Health Care^85^	Harvard University	Gain understanding of fundamental concepts related to AI, emerging modern methods, and potential roles of AI across components of health care industry.
Designing and Implementing AI Solutions for Health Care^86^	Harvard University	Gain understanding of key technical concepts of emerging AI methods including deep learning, reinforcement learning, interpretable and explainable AI techniques, frameworks for AI method development pipeline and real-world implementation as well as executions.
Medical Informatics Training Programs^87^	National Library of Medicine	Gain knowledge and experiences in computational biology using novel methods to better understand biological systems and computational health sciences focusing on novel computational methods for clinical and health data standards.

*Abbreviation*: AI: artificial intelligence.

## Data Availability

No data were analyzed or generated for this article.
